# Bacterial membranes are the target for antimicrobial polysiloxane-methacrylate copolymer

**DOI:** 10.1007/s10856-016-5669-6

**Published:** 2016-01-19

**Authors:** Joanna Jońca, Cecylia Tukaj, Władysław Werel, Urszula Mizerska, Witold Fortuniak, Julian Chojnowski

**Affiliations:** Chair & Department of Pharmaceutical Microbiology, Faculty of Pharmacy with Subfaculty of Laboratory Medicine, Medical University of Gdańsk, Al. Gen. J. Hallera 107, 80-416 Gdańsk, Poland; Centre of Molecular and Macromolecular Studies, Polish Academy of Sciences, Sienkiewicza 112, 90-363 Lódź, Poland; Department of Electron Microscopy, Faculty of Medicine, Medical University of Gdańsk, Debinki 1, 80-211 Gdańsk, Poland

## Abstract

Antibacterial polysiloxane polymers with pending *tert*-butylamine groups are a novel class of compounds that are compatible with silicone elastomers, but their mechanism of action is not well understood. The research into their action mechanism was conducted on a polysiloxane copolymer grafted with *tert*-butylaminoethyl methacrylate and covalently attached fluorescein. Fluorometric measurements results suggest that the polymer forms a stable link with bacteria. The results of β-galactosidase enzyme assay with the use of ortho-nitrophenyl-β-galactoside as a substrate show that the polymer has a damaging effect on bacterial membranes. The scanning and transmission electron micrographs of *Escherichia coli* cells incubated with the polymer prove further that the polymer’s site of action is bacterial cell membranes. In order to investigate the polymer interaction with bacterial membranes the fluorescein labelled polymer was incubated with bacterial cells and membranes isolation and identification method was next applied. The *E. coli* membrane fractions were identified by light scattering, protein content, oxidase NADH activity and *N*-phenylnaphtylamine fluorescence measurements, as well as electron microscopy. Oxidase NADH and *N*-phenylnaphtylamine were the inner membrane markers. The bacterial membranes were then tested for the presence of the polymer. The experiments gave evidence that the copolymer binds to the inner bacterial membrane. Further studies, where the copolymer was incubated with isolated mixed (inner and outer) membrane fractions, proved that the copolymer exerts more destructive effect on *E. coli* outer membrane. The damaging effect on the membranes is concentration dependent.

## Introduction

A growing resistance to antibiotics observed in many microbes is a serious concern of modern medicine. Resistant strains are often the cause of nosocomial infections and increase the cost of treatment [1–6]. Emerging resistance of bacteria to disinfectants also becomes an increasing threat [7–10]. In addition, biocorrosion, biofouling and biodegradation are a major problem in industry [11, 12]. Polymers with antimicrobial properties are an attractive alternative to commonly used disinfectants and have many potential applications in medicine and industry discussed in many reviews [13–19].

Antimicrobial polysiloxane polymers, because of their unique properties and high antimicrobial activity, are of special interest in this field. Polysiloxane polymers with quaternary ammonium, imidazolium and other groups, as well as polysilsesquioxanes with quaternary ammonium groups were shown to be active against Gram-positive and Gram-negative bacteria [20–23]. It is known that biocidal activity of polysiloxane polymers is influenced by such properties as the type of biocidal groups attached to the polymer chain, the density of antimicrobial groups on the polymer, alkyl chain length and the structure of the counterion [21–24]. It is not clear how those polymers cause a damaging effect although it was suggested that the action mechanism of polysiloxane polymers with pending quaternary ammonium salt (QAS) groups is via interactions with bacterial membranes in a manner similar to low molecular weight QASs [21]. It was found that in case of the polymers with pending QAS groups an optimal length of n-alkyl chain at nitrogen (C8) is necessary for the greatest bacterial activity. The dependence of the antimicrobial activity on hydrophobicity seems to support the hypothesis that the activity of QAS substituted polysiloxane polymers depends on interactions with bacterial membranes.

A high cationic charge in case of polymers with ammonium and imidazolium groups lowers their compatibility with hydrophobic materials such as silicones. Recently, it was shown that methacrylate polymers with *tert*-butylamine groups are potent antimicrobials [25]. Therefore, polysiloxane polymers with uncharged pendant *tert*-butylamine groups would be especially attractive additives to silicone elastomers. Additionally, grafting such polymers with polymethacrylate further broadens their spectrum of possible applications. They may find wide applications in medicine and industry, especially as additives to silicone materials and methacrylate paints [23].

There is still a question as of the mechanism of action of those polymers. It was shown that the activity of poly[2-(*tert*-butylamino)ethyl methacrylate] polymer (PEB-b-PTBAEMA) depends on the presence of Ca^2+^ ions [25]. It might suggest that those polymers act on the outer membrane lipopolysaccharide (LPS) of Gram negative bacteria. The polysiloxane polymers with similar groups may therefore also interact with bacterial outer membrane. However, it was yet not investigated. The insolubility of those polymers makes the investigation of their bacterial activity difficult as it is only possible in two-phase systems. Therefore, water-soluble analogues were synthesised with pending *tert*-butylethylammonium groups. The aim of this study is to give an insight into the target site of polysiloxane-methacrylate copolymers with pending *tert*-butylethylammonium groups.

## Materials and methods

**Fig. 1:**
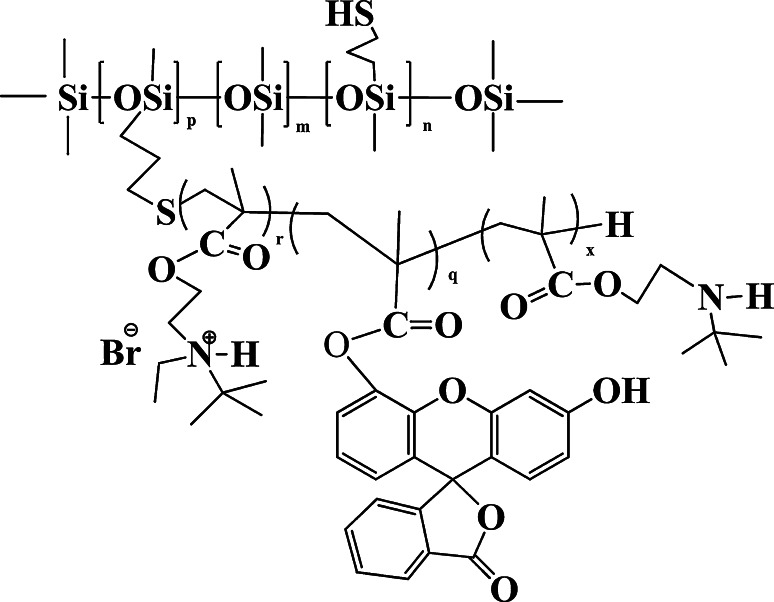
The structure of {poly[(3-mercaptopropyl)methylsiloxane-*co*-dimethylsiloxane]-graft-oligo-[2(*N*,*N*-*tert*-butylethylammonio)ethyl methacrylate bromide]} with attached fluorescein; m = 128, p = 8, n = 18, r + q + x = 5, (r = 3), M_n_ = 2.3 × 10^4^ g/mol

The studied polymers were a poly[(3-mercaptopropyl)methylsiloxane-*co*-dimethylsiloxane]-graft-poly(2-*tert*-butylaminoethyl methacrylate) copolymer after the reaction with ethyl bromide and the same copolymer with covalently attached fluorescein group to methacrylate chain (Fig. 1). Both copolymers were synthesized by the method of the free radical polymerization of 2*N*-*tert*-butylaminoethyl methacrylate in the presence of poly[(3-mercaptypropyl)methylsiloxane-*co*-dimethylsiloxane]. Chain transfer to mercaptyl groups led to the grafting of polymethacrylate chain to the siloxane copolymer. The obtained graft copolymer was subjected to the Mienshutkin reaction with ethyl bromide [23]. The fluorescein labelled graft copolymer was prepared by the addition of 5 mol% of fluorescein methacrylate (97 % declared purity) to the methacrylate monomer used in the chain transfer polymerization. The stock solutions of tested polymers were prepared in distilled water and stored at 4 °C.

The bacteria strains used were *Escherichia coli* (ATCC 8739), *Proteus vulgaris* (NCTC 4635), *Pseudomonas aeruginosa* (ATCC 9027), *Staphylococcus aureus* (ATCC 6530) and *Enterococcus hirae* (ATCC 10541). The bacteria were activated from frozen glycerol stocks and then stored on LB or BHI agar medium at 4 °C.

Fluorescein methacrylate and all chemical reagents for the synthesis of the polymer, ortho-nitrophenyl-β-galactoside (ONPG), Hepes, and reagents for electron microscopy were purchased from Sigma-Aldrich (St. Louis, MO, USA). All chemicals were of analytical reagent grade. The culture media were purchased from BD (Franklin Lakes, NJ, USA). Epon 812 was purchased from Merck (Darmstadt, Germany).

### Antimicrobial Activity Assay

The antibacterial tests were performed against *E. coli* (ATCC 8739), *P. vulgaris* (NCTC 4635), *P. aeruginosa* (ATCC 9027), *S. aureus* (ATCC 6530) and *E. hirae* (ATCC 10541). The minimum inhibitory concentration (MIC) values were determined by a standard microdilution technique as described by Mizerska et al. [23]. Overnight bacterial cultures were regrown to mid-logarithmic phase in Mueller–Hinton broth, diluted to the density of approximately 1 × 10^5^ CFU/ml and dispensed into microtiter plate wells. The plates were incubated at 37 °C for 24 h and the growth of bacteria was determined. The Minimum Inhibitory Concentration (MIC) value was taken as the lowest concentration of the polymer that inhibits visible growth of bacteria. To determine the Minimum Bactericidal Concentration (MBC) value after 24 h of incubation with the polymer bacterial suspension was spread on the MH agar. The agar plates were incubated at 37 °C for the next 24 h and then examined for visible growth of bacterial colonies.

### Assessment of polymer affinity to bacterial cells

The polysiloxane-methacrylate copolymer with covalently attached fluorescein probe was used in the experiment. Overnight *E. coli* culture was regrown to the mid-logarithmic phase (OD_600_ = 0.5–0.6) in LB broth with vigorous shaking at 37 °C. Bacterial cells were centrifuged at 10,000×*g* for 1 min in MPW-50 centrifuge (MPW Med. Instruments, Krakow, Poland) and suspended in PBS buffer (pH 7.4) at OD_600_ = 0.6. The polymer was added to bacterial suspension to the final concentration of 12 mg/ml. Negative and positive controls were prepared by using PBS buffer in place of polymer and bacterial cells suspension respectively. After 45 min of incubation at room temperature the fluorescence was measured with microplate reader Infinite M200 Pro (Tecan, Männedorf, Switzerland). Excitation and emission wavelengths were set at λ = 480 nm and λ = 535 nm respectively. In the next step, the bacterial suspension was centrifuged for 1 min. The supernatant was carefully removed using a pipette and the remaining cell pellet was resuspended in PBS buffer. The fluorescence of supernatant and bacterial suspension was measured as previously and then the steps of centrifugation and resuspension of bacterial cells were repeated for the second and third time.

### Fluorescence microscopy

The polysiloxane-metacrylate copolymer with attached fluorescein probe was used in the experiment. Overnight *E. coli* culture was regrown to the mid-logarithmic phase (OD_600_ = 0.5–0.6) in LB broth with vigorous shaking at 37 °C. Bacterial cells were centrifuged at 10,000×*g* for 1 min in centrifuge MPW-50 (MPW Med. Instruments, Krakow, Poland) and suspended in PBS buffer (pH 7.4) to OD_600_ = 0.1. The polymer was added to bacterial suspension to the final concentration of 15 mg/ml. The suspension was incubated for 45 min at room temperature, diluted and then placed on a glass slide. The samples were examined with laser scanning microscope Nikon TE-300 (Nikon, Tokyo, Japan) with Cooled Digital Camera (C4742-95, Hamatsu, Hamamatsu City, Japan), and filter cube Nicon B2A. The acquisition software was Lucia Image (Laboratory Imaging s.r.o., Praha, Czech Republic).

### Inner membrane permeability assessment

The permeation of *E. coli* inner membrane was determined by the method of Ibrahim et al. [26]. The measurement of ortho-nitrophenol production was performed using ortho-nitrophenyl-β-galactoside (ONPG) as a substrate. Overnight bacterial culture was regrown at 37 °C to mid-logaritmic phase (OD_600_ = 0.5–0.6) in LB broth supplemented with 2 % lactose. Bacterial cells were collected by centrifugation at 10,000×*g* for 1 min in centrifuge MPW-50 (MPW Med. Instruments, Krakow, Poland), washed and resuspended in PBS buffer (pH 7.4). Bacterial suspension was transferred into the wells of a microtiter plate, followed by ONPG to final concentration of 1.5 mM. Proper dilutions of polymer (to final concentrations of 0.05, 0.5, 1, 2, 4, 8 mg/ml) in PBS buffer were then added to the wells. The plates were incubated with a gentle rocking at 37 °C. Positive and negative controls were also prepared. Ethylenediaminetetraacetic acid (EDTA) to the final concentration of 10 mM was added in the positive control, gentamycin to the final concentration of 20 μg/ml and distilled water were added in place of the polymer in the negative controls. The measurement of ortho-nitrophenol production over time was monitored using microplate reader Infinite M200 Pro (Tecan, Männedorf, Switzerland) at 415 nm.

### Scanning Electron Microscopy

*Escherichia coli* cells were suspended in 1 ml of PBS buffer (pH 7.4) to OD_600_ = 0.1 and incubated with 10 mg/ml polymer for 2 h at room temperature. The cells were centrifuged at 10,000×*g* for 1 min and resuspended in 1 ml 10 mM Hepes buffer (pH 7.4). The cells were then fixed with 2.5 % glutaraldehyde for 2 h at 4 °C, washed three times with the buffer, and then placed on a 0.1 % poly-l-lysine coated glass slides. The samples were then prepared by a standard procedure as described by Codling et al. [27], and examined by scanning electron microscopy (SEM). Samples were coated with a fine gold layer (about 200 μm thick) using the ion coating JEOL JFC 1200 apparatus (Jeol, Tokyo, Japan). SEM images were taken with the Jeol JSH 5500 LV (Jeol, Tokyo, Japan) microscope in high vacuum mode at the acceleration voltage of 10 kV.

### Transmission Electron Microscopy

*Escherichia coli* cells were suspended in 1 ml of PBS buffer (pH 7.4) to OD_600_ = 0.1 and incubated with the polymer (2.5 mg/ml) for 2 h and 24 h at room temperature. The cells were centrifuged at 10,000×*g* for 1 min, fixed overnight in 6.25 % glutaraldehyde in 0.1 % cacodylate buffer pH 7.4 at 4 °C, and then fixed in 2 % osmium tetroxide in the same buffer. Samples were dehydrated, embedded in Epon 812, sectioned and then stained using uranyl acetate and lead citrate. The bacteria samples were examined with transmission electron microscope Jeol JEM 1200EX II (Jeol, Tokyo, Japan) at voltage 80 kV.

### Membranes isolation

*Escherichia coli* membranes were isolated by the modified procedure of Kucharczyk et al. [28]. *E.coli* ATCC 8753 cells were transferred from agar medium to fresh LB broth and incubated overnight with vigorous shaking at 37 °C. 4 ml of cell culture was then transferred to four tubes with 250 ml LB medium each, and incubated as previously at 37 °C to OD_600_ = 0.6. 250 ml frozen on ice 10 mM Tris–HCl was then added to each tube and cells were harvested by centrifugation in K70D (MLW, Engelsdorf, Germany) centrifuge at 3500xg for 30 min at 4 °C. The cell pellet was resuspended in 2 ml cold 200 mM Tris–HCl buffer pH 7.4. Then, 2 ml cold 1 M sucrose-200 mM Tris–HCl and 70 μl lysozyme (12 mg/ml) were added to the suspension. The tubes were incubated on ice for 5 min. Then, 4 ml of cold water was added to each tube, followed by 20 μl 200 mM phenylmethylsulfonyl fluoride (PMSF) and 20 μl 1 M dithiothreitol (DTT). The mixture was then incubated on ice for 10 min. Spheroplasts where then lysed by sonification (6 min, 30 % pulsation, 50 % amplitude) at 0 °C using sonificator Sonoplus (Bandelin, Berlin, Germany). Lysates were centrifuged for 20 min at 5000×*g* at 4 °C to remove intact cells. Step sucrose gradients were prepared by layering 10 ml 17 % sucrose over 5 ml 55 % sucrose. The sucrose solutions were prepared in 10 mM Tris–HCl pH 7.4 buffer with 3 mM EDTA pH 7.4. The lysed bacterial suspension was layered on top of the gradient and centrifugation was carried out for 45 min in ultracentrifuge L7, rotor SW28 (Beckman, Brea, CA, USA) at 24,000 rpm at 4 °C. A “crude” membrane fraction (about 2.5 ml) was collected from the intermediate of 55 and 17 % sucrose. Equal amount of 3 mM EDTA, and then 10 μl 1 M DTT and 10 μl 200 mM PMSF were added to the membrane fraction. It was then layered on top of six-step gradients containing 3 ml 55 % sucrose, 6 ml 50 % sucrose, 6 ml 45 % sucrose, 6 ml 40 % sucrose, 5 ml 35 % sucrose and 4 ml 30 % sucrose, and centrifuged for 16 h in ultracentrifuge L7, rotor SW28 (Beckman, Brea, CA, USA) at 24,000 rpm at 4 °C. All sucrose solutions contained 10 mM Tris–HCl pH 7.4 and 3 mM EDTA pH 7.4. Gradient fractions (370 µl each) were collected to a microtiter plate.

### Analyses of membrane fractions

Membrane fractions were analysed for light scattering at λ = 450 nm or λ = 600 nm in microtiter plates reader Infinite M200 Pro (Tecan, Männedorf, Switzerland). For the analyses of refraction index and sucrose density in refractometer (Zeiss, Oberkochen, Germany) sucrose gradient was prepared with 5 ml 15 % sucrose in 3 mM EDTA instead of “crude” membrane fraction. Protein content in each fraction was measured by Bradford assay according to the procedure of Bradford reagent supplier (Sigma-Aldrich, St. Louis, MO, USA).

### Oxidase NADH activity assay

The assay was carried out according to the procedure described by Osborn et al. [29]. Incubation mixtures contained 300 μl 12.5 mM Tris–HCl pH 7.4, 20 μl membrane fractions and 5 μl 36 mM NADH in 50 mM Tris–HCl pH 7.4. The rate of decrease in absorbance at λ = 340 nm (Δabs./min) was measured in microtiter plates reader Infinite M200 Pro (Tecan, Männedorf, Switzerland) at 24 °C.

### Measurement of *N*-phenylnaphtylamine (NPN) fluorescence

1 ml 30 mM NPN was added to spheroplasts suspension before sonification step. The membrane isolation procedure was carried out as previously and fractions were analyzed for fluorescence (excitation wavelength λ = 350 nm; emission wavelength λ = 405 nm) in microtiter plates reader Infinite M200 Pro (Tecan, Männedorf, Switzerland).

### Analyses of membrane fractions by transmission electron microscopy

Membrane fractions were analyzed by transmission electron microscopy according to the procedure described by Osborn et al. [30]. The membrane fractions were diluted in two volumes of 3 mM EDTA in 10 mM Tris–HCl pH 7.4 and centrifuged for 2 h in ultracentrifuge L7, rotor SW28 (Beckman, Brea, California) at 24000 rpm at 4 °C. The pellet was fixed overnight in 6.25 % glutaraldehyde in 0.1 % cacodylate buffer pH 7.4 at 4 °C, and then fixed in 2 % osmium tetroxide in the same buffer. The further procedure of preparing the samples (fixation, dehydration and staining) was carried out according to the method described in ‘Transmission Electron Microscopy’ section.

### Assessment of polymer affinity to bacterial membranes

100 μl 80 mg/ml polymer suspension was added to the *E. coli* culture (final polymer concentration 33 µg/ml). After 15 min incubation bacteria were centrifuged, the membrane isolation procedure was then carried out and fractions were analyzed for fluorescence (excitation wavelength λ = 480 nm; emission wavelength λ = 535 nm) in microtiter plates reader Infinite M200 Pro (Tecan, Männedorf, Switzerland). In other experiments the polymer (1.2 mg/ml and 4 mg/ml) was also added to the “crude” membrane fractions and then the membranes were fractioned as previously. All fractions were also analyzed for light scattering at λ = 450 nm, protein content and oxidase NADH activity.

## Results

### Assessment of polymer affinity to bacterial cells

The results of preliminary antimicrobial activity assessment of water insoluble polysiloxane-methacrylate copolymer in two-phase system showed that the polymer is a potent antimicrobial (data not shown). However, it was impossible to determine the polymer’s site of action. A water soluble polysiloxane-methacrylate copolymer with pending *t*-butylethylammonium groups and its analogue with attached fluorescein groups (Fig. 1) were therefore used for this purpose. The antimicrobial activity assay for those polymers was conducted by determination of minimum inhibitory concentration (MIC) and minimum bactericidal concentration (MBC) values by a standard serial microdilution technique.

The studied polymers were active against Gram-positive and Gram-negative bacteria (Table 1). Overall, the polymers were more active against Gram-positive bacteria than Gram-negative bacteria with the exception of *E. coli* against which both polymers showed a good activity (MIC = 60 μg/ml). The polymers were the most active against *E. hirae* (MIC = 30 μg/ml) and *S. aureus* (MIC = 60 μg/ml). The polymers were the least active against *P. aeruginosa* (MIC = 2.4 mg/ml) and *P. vulgaris* (MIC > 15 mg/ml). The polymer with fluorescent probe showed a similar activity to the base polymer. The MBC values were similar to MIC values for both polymers.

**Table 1:** Minimum inhibitory concentration (MIC) and minimum bactericidal concentration (MBC) of polysiloxane-methacrylate copolymer and polysiloxane-methacrylate copolymer with fluorescein group

Bacterial strain	Polysiloxane-methacrylate copolymer		Fluorescein labelled polysiloxane-methacrylate copolymer	
	MIC (μg/ml)	MBC (μg/ml)	MIC (μg/ml)	MBC (μg/ml)
*E. coli* ATCC 8739	60	60	30	30
*P. vulgaris* NCTC 4635	>15,000	>15,000	3350	3350
*S. aureus* ATCC 6530	60	60	60	120
*E. hirae* ATCC 10541	30	30	30	30
*P. aeruginosa* ATCC 9027	240	240	60	240

**Fig. 2:**
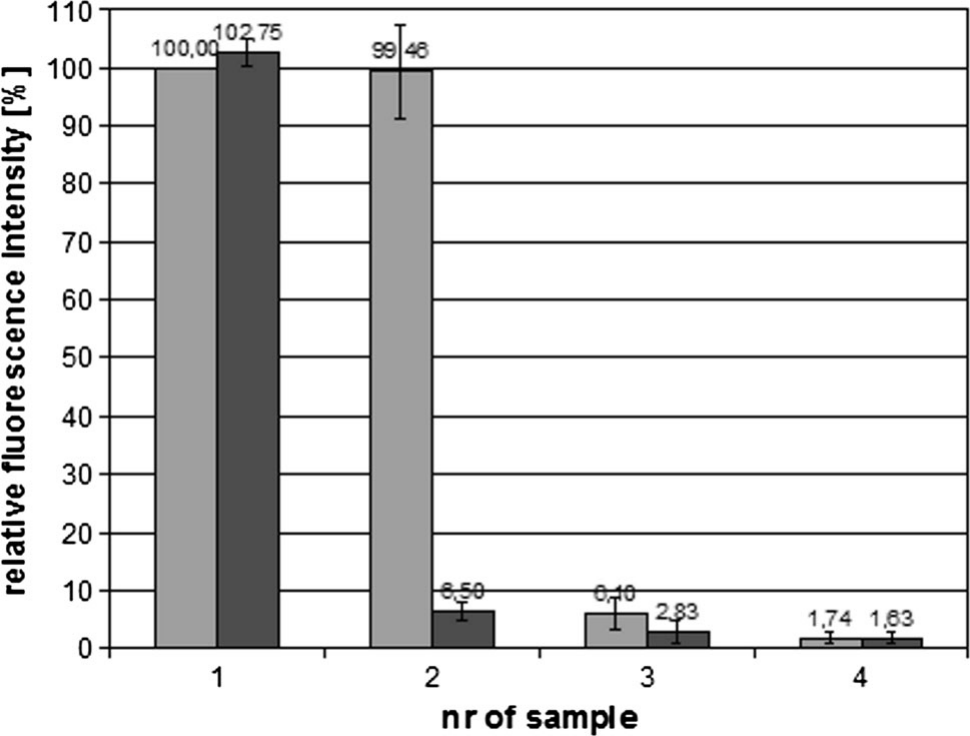
Assessment of the polymer affinity to bacterial cells. The graph shows the relative fluorescence of polymer solutions: 1-polymer solution (12 mg/ml) and bacterial suspension with the polymer (*grey and black bar* respectively); 2,3,4-supernatant and bacterial suspension (*grey and black bar* respectively) after first, second and third centrifugation; (n = 6)

**Fig. 3:**
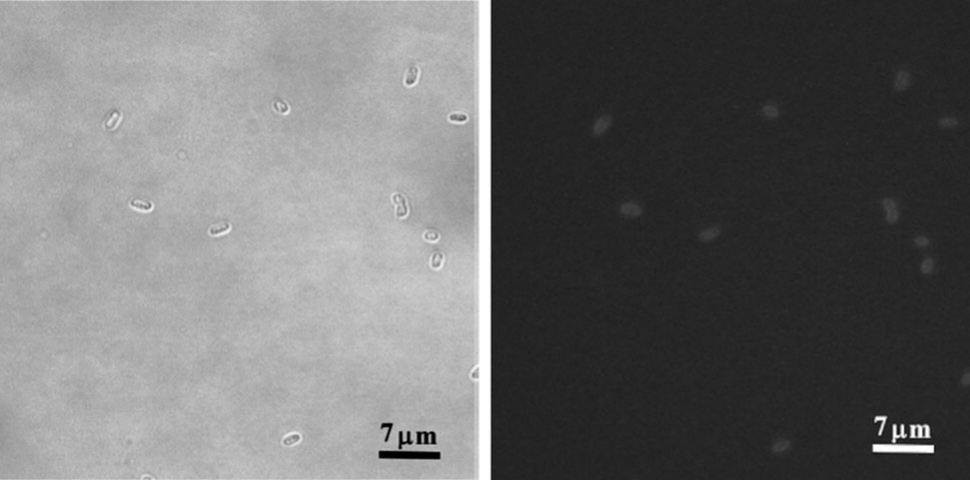
Visible-light microscopy and fluorescence microscopy of *E. coli* cells treated with the polysiloxane-methacrylate copolymer labelled with fluorescein. The cells in mid-logarithmic phase were incubated with 15 mg/ml polymer solution

In order to investigate the interactions of the polymer with bacterial cells and its binding site a polysiloxane-methacrylate copolymer with fluorescein attached group was used. The binding of the polymer to *E. coli* cells was determined by incubation of bacterial suspension with the polymer, and then removal of unbound polymer by centrifugation. The cell pellet was resuspended in a new portion of PBS buffer, and subsequently fluorescence of supernatant and bacterial suspension was measured. As the reference the fluorescence of polymer solution at the same concentration was used (the first grey bar on Fig. 2). The “washing” steps and measurement of fluorescence were repeated three times. The results were showed as a percentage of base polymer solution fluorescence (Fig. 2). After the second cycle of centrifugation bacterial cells suspension showed 2.8 % of base fluorescence and even after the third cycle it retained about 1.6 % of base fluorescence. The experiment gave the evidence that the polymer exhibits a high affinity to bacterial cells. It allowed to infer that the polymer would remain attached to the cells during microscope slides preparation and isolation of cell constituents. It was therefore possible to identify the polymer’s site of attachment. For this purpose, *E. coli* cells were incubated with fluorescein labelled polymer and visualized with fluorescence microscopy (Fig. 3). Bacterial cells were fluorescent in the presence of the polymer which confirms that the polymer shows affinity towards bacterial cells. The fluorescence intensity was the highest at the boundaries of the cells which further suggests that the polymer attaches mainly to the cell surfaces.

### The analyses of changes in cell structure caused by the polymer

**Fig. 4:**
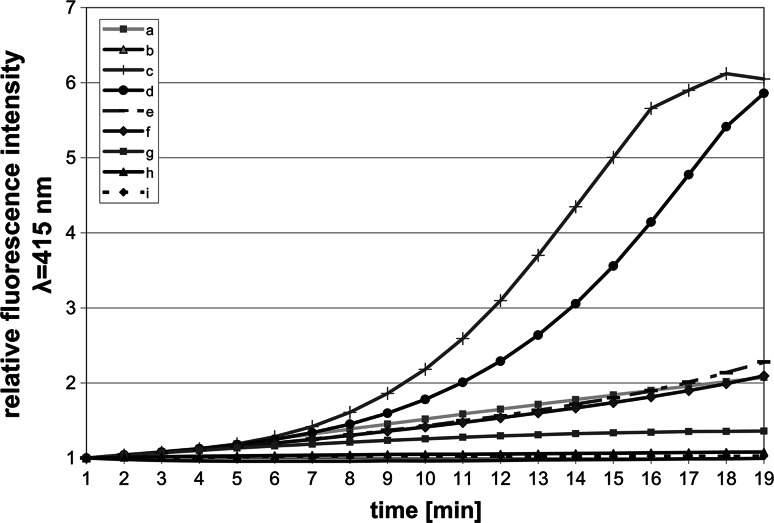
Permeation effect on the inner membrane of *E. coli* cells incubated with polysiloxane-metacrylate copolymer. Damaging effect on the membrane was determined by the measurement of ortho-nitrophenol absorbance at λ = 415 nm. The samples contained: *a* 10 mM EDTA (positive control); *b* 20 μg/ml gentamycin; *c* 8 mg/ml polymer; *d* 4 mg/ml polymer; *e* 2 mg/ml polymer; *f* 1 mg/ml polymer; *g* 0.5 mg/ml polymer; *h* 0.05 mg/ml polymer; *i* negative control

The above experiments suggested that the lethal effect of the polymer is the result of its binding to bacterial cells, possibly to bacterial membranes. The ability of the studied polymer to permeate *E. coli* inner membrane was evaluated by the measurement of conversion of the substrate (ONPG) into ortho-nitrophenol. The substrate became accessible to intercellular β-galactosidase after destruction of the inner membrane. The permeability effect on the membrane was dose dependent within polymer concentrations of 0.05–8 mg/ml (Fig. 4). The effect of EDTA, which was used in control, was comparable to the effect of 2 mg/ml polymer. Gentamycin at 20 μg/ml concentration had no effect on inner membrane permeability.

**Fig. 5:**
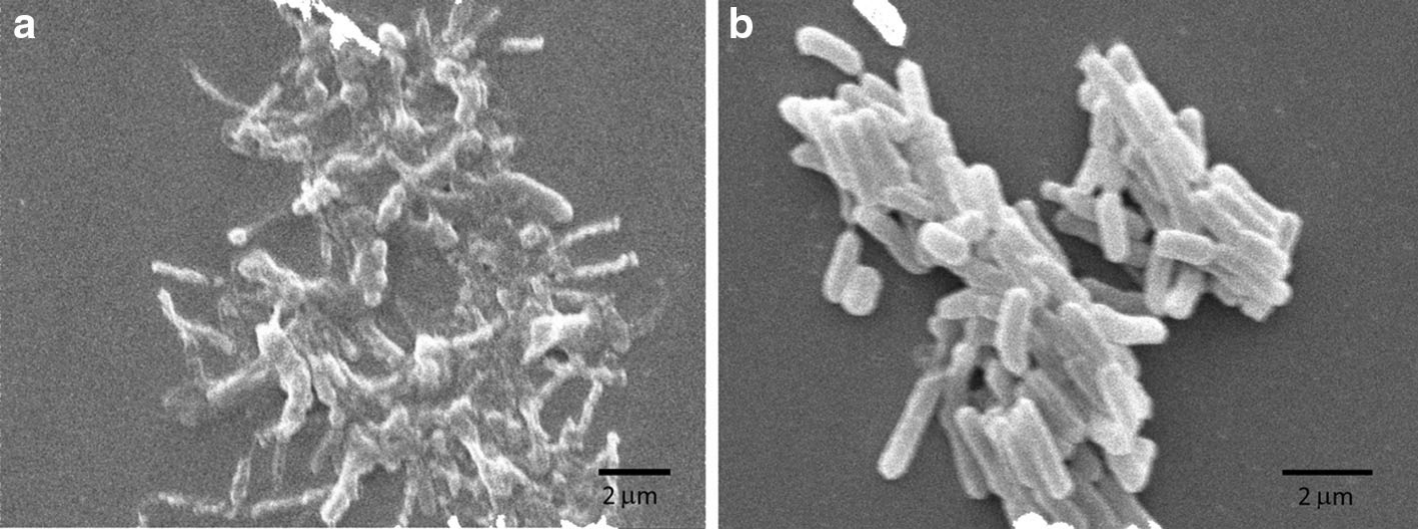
Scanning electron microscopy of *E. coli* cells treated with 10 mg/ml polymer (**a**) and control without addition of the polymer (**b**)

The SEM images of *E. coli* cells incubated with the polymer revealed many structural changes in cells morphology (Fig. 5). The cells were wrinkled and shrank in comparison to control where they were smooth and intact. Their contours became distorted and in some cases blebs formed in membranes.

**Fig. 6:**
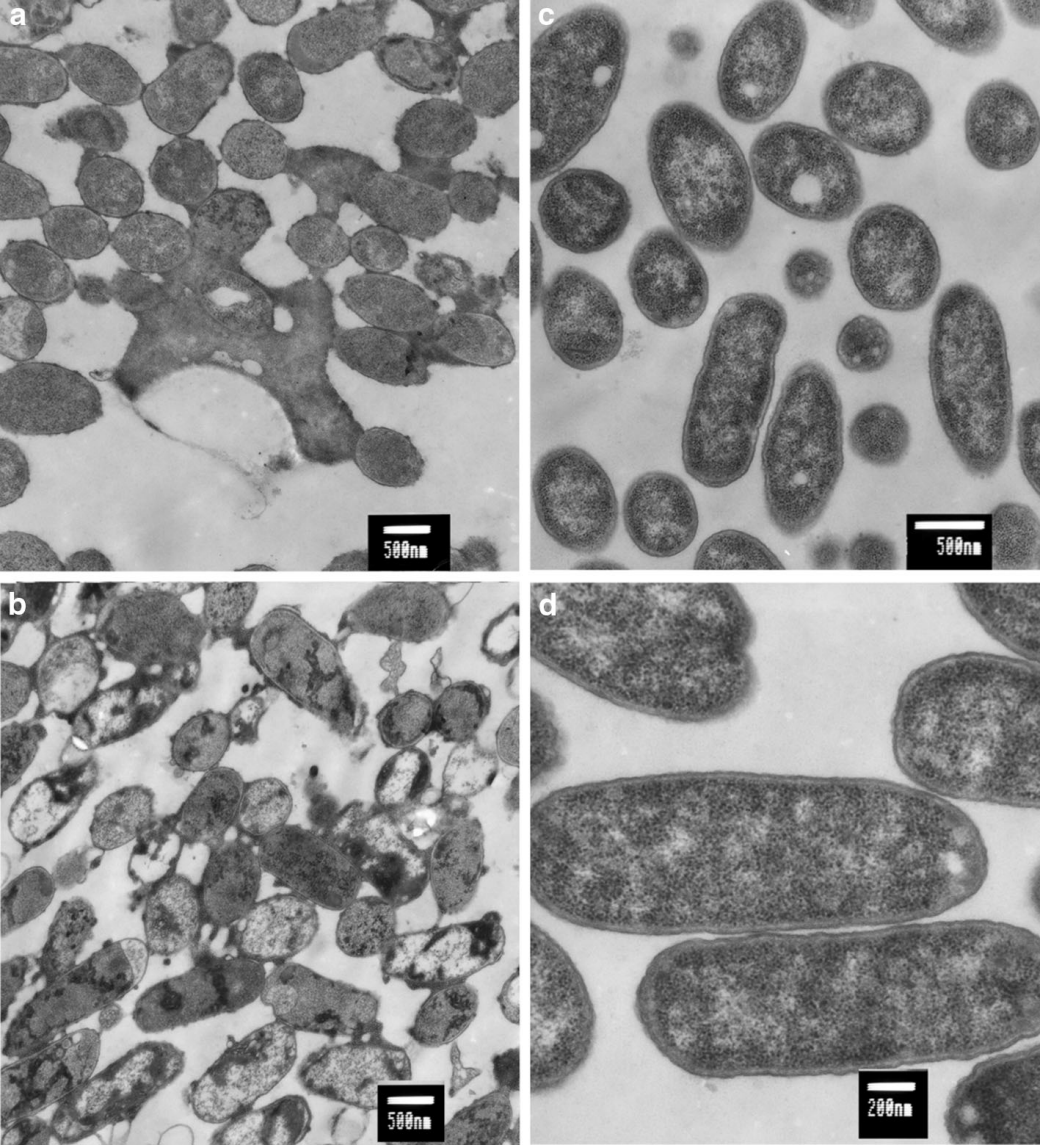
Transmission electron microscopy of *E. coli* cells treated for 2 h (**a**) and 24 h (**b**) with 2.5 mg/ml polymer. The controls without the addition of the polymer after 2 h (**c**) and 24 h (**d**) incubation

The examination by transmission electron microscopy of thin sections of *E. coli* cells treated with the polymer revealed structural and morphological changes in the cells (Fig. 6). The polymer’s action led to formation of blebs and indentations in cell membranes, and subsequent leakage of cells content. In some cases, the membranes became separated from the cells. A granular dark material was additionally visible inside of the cells. All those changes became visible after 2 h of incubation with the polymer, and became more prominent after 24 h of incubation.

### Analyses of polymer binding to bacterial membranes

**Fig. 7:**
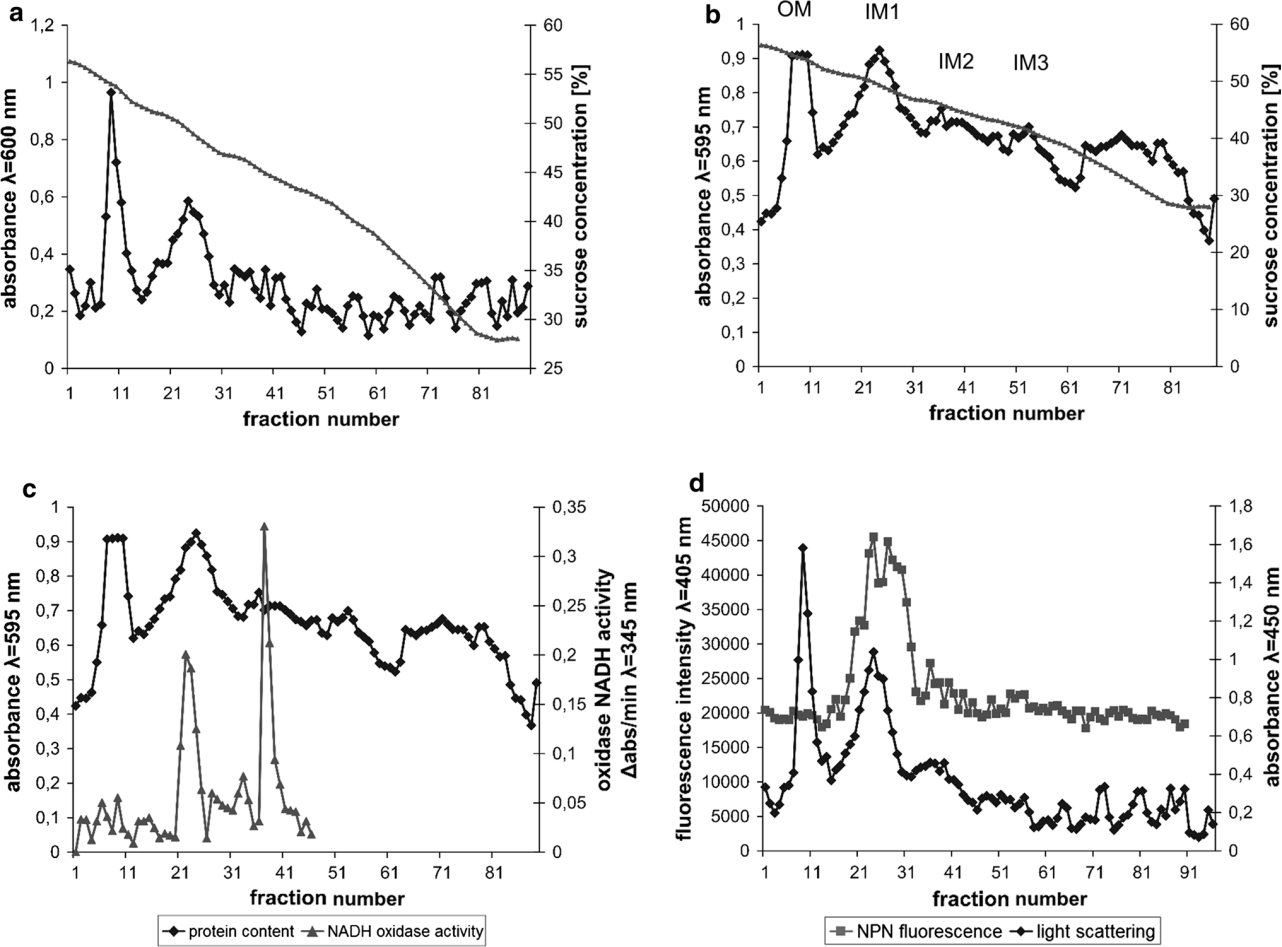
Membrane fractions obtained by centrifugation of “crude” membranes in six-step sucrose gradient. The fractions were analysed for light scattering at λ = 600 nm (**a**), protein content measured with Bradford assay (**b**), protein content and NADH oxidase activity (**c**), and light scattering at λ = 450 nm and NPN fluorescence at λ = 405 nm (**d**). OM and IM symbols in *panel* (**b**) indicate outer membrane and inner membrane fractions respectively

Prior to the examination of the polymer binding to *E. coli* membranes the method of isolation and identification of bacterial membranes was applied and optimized. After lysis of spheroplasts, the mixture of membrane fractions and cytoplasmic constituents was centrifuged in two-step sucrose gradient (17 and 55 %) in order to obtain a “crude” membranes fraction. The membranes were then separated in six-step sucrose gradient on outer and inner membranes. Two bands of different buoyant density were obtained which strongly scattered light. The contents of the tube were divided on fractions which had different light-scattering properties and protein content (Fig. 7a, b).

Two dominant membrane bands were visible, tentatively identified as outer and inner membranes, located in 53–51 % sucrose (OM) and 48–46 % sucrose (IM1). In addition to the main inner membrane fraction (IM1), two additional distinct subfractions were visible in 46–41 % sucrose (IM2) and 43–38 % sucrose (IM3), which had weaker light-scattering capability and lower protein content.

In order to further identify the membranes fractions the activity of NADH oxidase, an enzyme bound with the inner membrane of *E. coli*, was measured in each fraction. The peaks of NADH oxidase activity were located mainly in the IM bands with the highest activity in IM2 band (Fig. 7c). A residual activity of the enzyme was also observed in an OM band.

The identification of membrane fractions was also performed with the use of *N*-phenylnaphtylamine (NPN). This compound shows increase in fluorescence after binding to inner membrane phospholipids and therefore was used as inner membrane marker. In this experiment bacterial culture was incubated with NPN and then membranes were isolated. The peak of NPN fluorescence was located mainly in the IM1 band (Fig. 7d). The analyses of oxidase NADH activity in membrane fractions as well as NPN fluorescence measurement definitely show that the fraction of higher buoyant density is the outer membrane fraction (OM) and the fractions of lower density (IM1, IM2 and IM 3) are the inner membrane fractions.

Membrane fractions (OM and IM1) were also visualized with the use of transmission electron microscope (data not shown). These fractions contained closed vesicles of different diameter. The OM band vesicles were smaller (0.1 μm diameter) than the IM1 band vesicles (0.05–5 μm). In the OM band could be observed coiled and C shaped structures characteristic to the outer membrane, whereas the IM1 band consisted mainly of uniform closed vesicles.

**Fig. 8:**
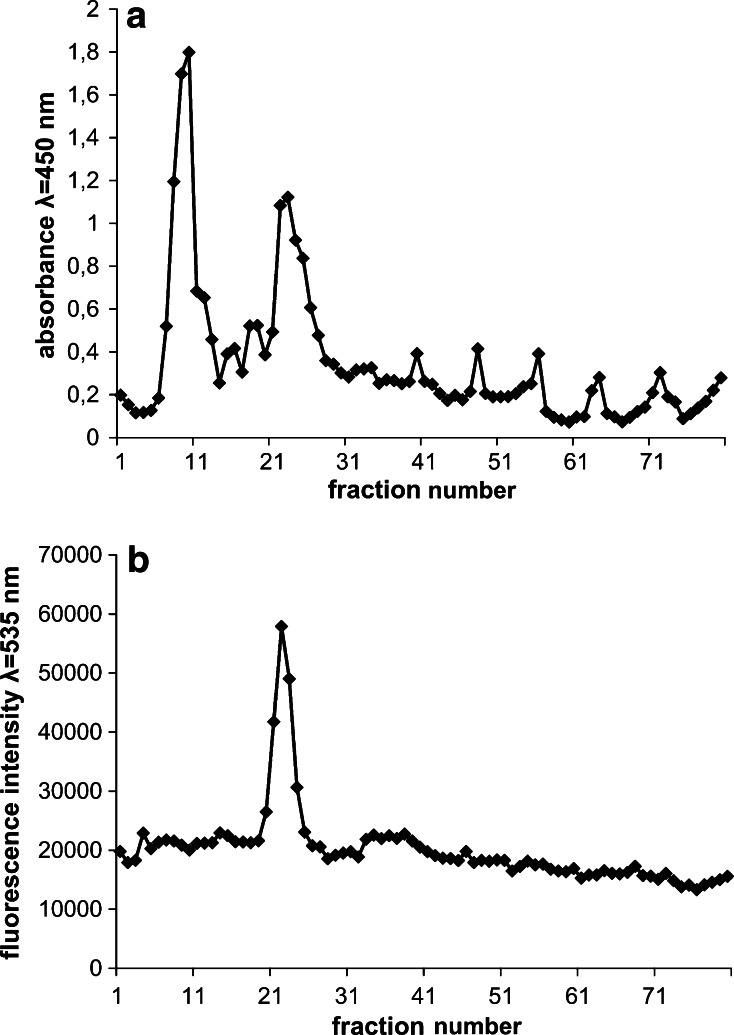
Membrane fractions obtained after 15 min incubation of *E. coli* culture with 33 μg/ml fluorescein-labelled polymer examined for light scattering λ = 450 nm (**a**) and fluorescence at λ = 535 nm (**b**)

Having optimized the techniques of isolation and identification of the bacterial membranes, in order to directly examine if the polymer interacts with *E. coli* membranes, isolation and identification of membrane fractions were carried out after incubation of *E. coli* cells with fluorescein-labelled polymer (33 μg/ml). The polymer’s fluorescence peak was observed only in the inner membrane (IM1) band (Fig. 8). There was no visible change in gradient pattern in the presence of the polymer in comparison to control (Fig. 7).

**Fig. 9:**
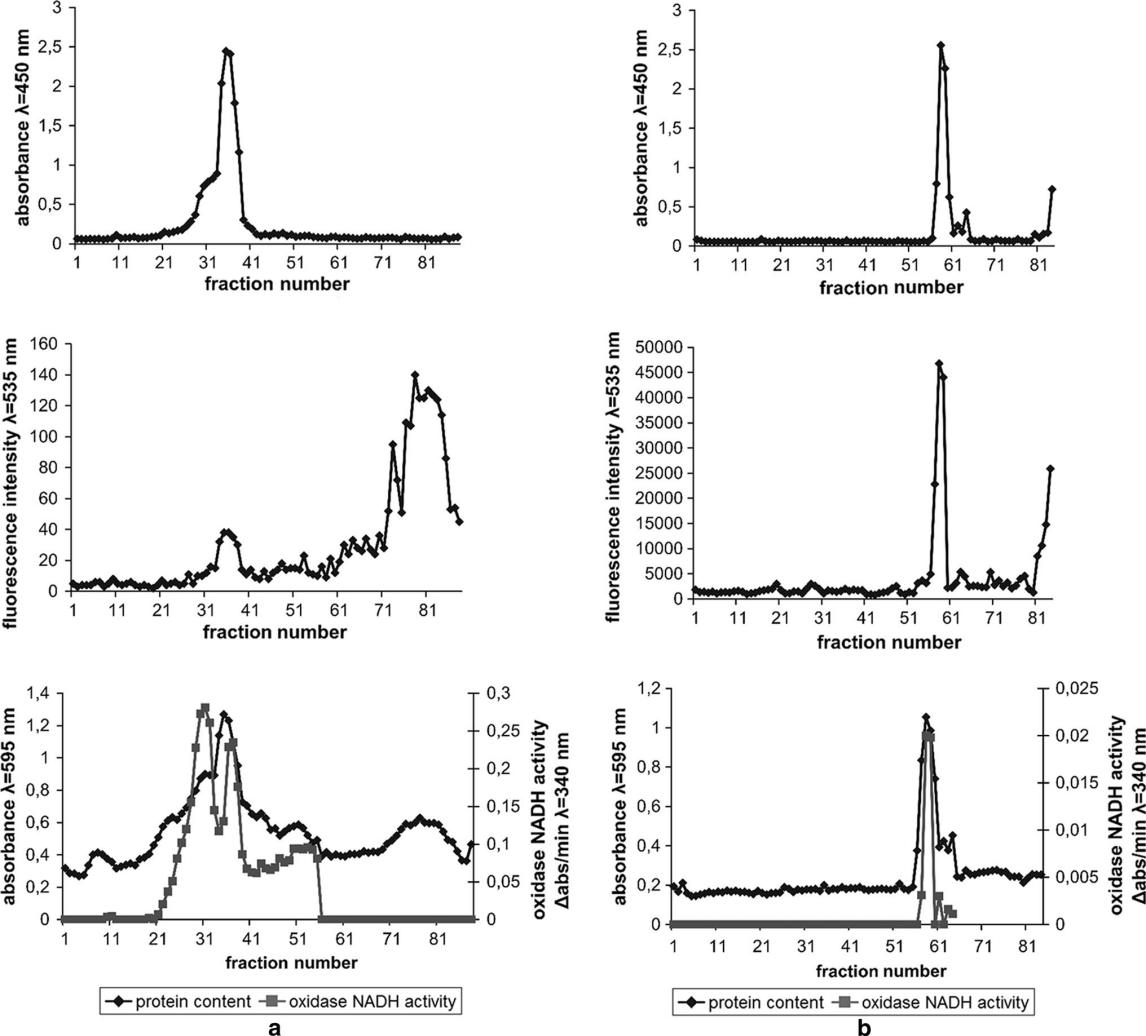
Membrane fractions isolated after incubation of “crude” membrane fractions with 1.2 mg/ml polymer (**a**) and 4 mg/ml polymer (**b**). The fractions were analysed for light scattering at λ = 450 nm (*top figures*), polymer fluorescence at λ = 535 nm (*middle figures*), protein content and oxidase NADH activity (*bottom figures*)

In the next experiment “crude” bacterial membranes were isolated in two-step sucrose gradient before addition of the polymer. The experiment was carried out in order to investigate the polymer’s affinity to inner and outer membranes as well as the damaging effect of the polymer on isolated membranes. In the experiment, two different concentrations of the polymer (1.2 and 4 mg/ml) were used. The incubation with the polymer (1.2 mg/ml) resulted in changed gradient patterns (Fig. 9) in comparison to control (Fig. 7). Analyses of light scattering (Fig. 9a) showed only a single band (fractions 27–31), which had an oxidase NADH activity and corresponded to the inner membrane (IM1). In that band the fluorescent polymer was localized. The outer membrane band (OM) was invisible on the light scattering pattern. However a small, residual peak of protein content was visible in the position of outer membrane band. The results signify the destructive effect of the polymer on the outer membrane with a small effect on the inner membrane (the IM bands locations were the same as in control). However, the IM band also changed with the increased concentration of the polymer (4 mg/ml) and became shifted towards sucrose of lower concentrations (Fig. 9b). The polymer’s fluorescence was still localized only in the band containing inner membrane fractions. These fractions retained NADH oxidase activity. The results indicate different affinity and damaging effect of the polymer to each membrane.

## Discussion

The mechanism of action of antibacterial polymers is still not clear, although a growing number of reports aimed at investigating those polymers are being published recently [31–34]. However, polysiloxane polymers with antibacterial activity have not yet been studied extensively. Therefore, the aim of these studies was to investigate the target site of polysiloxane-methacrylate copolymers on bacterial cells. Those uncharged polymers are hydrophobic and thus compatible with silicones. However, the analysis of their action is difficult as in this case it involves studies in two-phase system of bacterial suspension above the surface of the polymer [23]. It does not allow to indicate the target of polymer action in bacterial cells. The use of water soluble polymer allows on the other hand an easy analysis of antibacterial activity in solution by defining the MIC value, as well as investigation of the mechanism of the polymer interaction with bacterial cells. For this purpose, water soluble poly[dimethylsiloxane-*co*-(3-mercaptopropyl)methylsiloxne]-graft-poly[2(*N*,*N*-*t*-butylethylammonioethyl methacrylate bromide)] copolymer was used, as well as its analogue with covalently attached fluorescein group. The antimicrobial activity of *t*-butyl substituted polysiloxane-methacrylate copolymers is related to the presence of *t*-butyl group, but the mechanism of biocidal action of the polymer seems to be rather complex [23]. Antimicrobial activity of water-soluble polysiloxane polymers depends both on the presence of charged *t*-butylethylammonium group and polysiloxane-methacrylate chain.

We showed that the antibacterial activity of the polysiloxane-methacrylate copolymer depends on its concentration, and bacterial strain (Table 1). The effect of the presence of fluorescein group on polymer’s activity was negligible. The polymer was more active against Gram-positive bacteria. The cell wall of Gram-positive organisms, despite consisting of multiple layers of peptidoglycan, has many pores, which possibly allows a passage of the polymer. In addition, they lack the protection which the outer membrane provides for Gram-negative bacteria. That property of the cell wall of Gram-positive bacteria was also recognized as the reason of their susceptibility to many biocides [35, 36]. The lower activity of the polymer against *P. vulgaris* and *P. aeruginosa* suggest that outer membrane structure may be a factor in the action of the polymer. It is believed that the higher Mg^2+^ content in *P. aeruginosa* outer membrane is responsible for their resistance to many disinfectants, e.g. QAS. Also *P. vulgaris* is resistant to many cationic biocides because it has a less acidic type of outer membrane lipopolysaccharide [10].

Incubation of bacterial cells with the polymer, with subsequent removal of unbound polymer by centrifugation and resuspension of the cell pellet in order to analyze the amount of bound polymer, allowed to assess the mode of interaction of the polymer with bacteria. The experiment showed that the polymer binds efficiently to bacterial cells. After extensive washing about 1.6 % of the polymer remains bound to bacterial cells (Fig. 2). It is therefore possible to identify the polymer’s target of action within *E. coli* cells. For this purpose, *E. coli* cell suspension was incubated with fluorescein labelled polysiloxane-methacrylate copolymer and then viewed under the fluorescence microscope. The bound polymer caused fluorescence of bacterial cells on microscopic preparations which further confirms that the tested polymer binds to bacterial cells. The fluorescence intensity was the highest at the borders of the cells. The results suggest that bacterial surface structures, possibly the membranes, are the polymer’s site of action.

To examine if the membranes are the target of the polymer β-galactosidase assay was performed. A cytoplasmic enzyme β-galactosidase release from the cells and subsequent measurement of ortho-nitrophenol production is a method which allows assessment of bacterial inner membrane damage [37, 38]. *E. coli* cells are cultured in a medium containing lactose in order to induce β-galactosidase expression. Upon damage of the membranes the enzyme is released from the cytoplasm and converts the substrate ONPG in the samples to ortho-nitrophenol which increases absorbance at 412 nm. The absorbance of the ortho-nitrophenol was the function of time and polymer concentration (Fig. 4). The results of the experiment when gentamycin was used suggest that damage to only the outer bacterial membrane, as it is in case of gentamycin [39], does not stimulate ortho-nitrofenol production. Therefore, the increase in absorbance during the incubation of bacteria with the polymer is a proof that the bactericidal effect of the polymer is the consequence of the inner membrane damage. EDTA, which acts on LPS by removal of divalent cations, damages also the inner membrane, causing the increase of ortho-nitrofenol absorption, which is similar to that of the polymer at 2 mg/ml concentration. Therefore, it was proved that the action of the polymer involves damage of both bacterial membranes, which leads to the release of cytoplasmic constituents and may be the reason of cell death.

The TEM and SEM images of *E. coli* cells treated with the polymer give further evidence that the tested polymer acts by damaging bacterial membranes (Figs. 5, 6). The formation of blebs, wrinkles and shredding of the membranes lead to the release of cellular contents. As the result the cells shrink and undergo lysis. Additionally, granular accumulates were visible inside of the cells which suggests that the polymer may act not only on the bacterial membranes but also lead to intercellular damage and aggregation of cellular content.

The above experiments suggested that the polymer interacts with bacterial membranes. In order to confirm that hypothesis it is necessary to directly prove the interaction of the polymer with bacterial membranes. It is therefore necessary to isolate the bacterial membranes and assess if the polymer is bound to any of the membranes. In order to identify the target site of the polymer the bacterial cells were incubated with the fluorescein-labelled polymer prior to membrane isolation. The identification of both, outer and inner *E. coli* membrane fractions was then necessary. The membranes fractions patterns (Fig. 7) were similar to those obtained by others [28, 29, 40, 41]. According to their results outer membrane band should be localized in 55–45 % sucrose solution whereas inner membrane band in 45–30 % sucrose. The obtained bands were therefore tentatively identified as an outer membrane (OM) and three subfractions of inner membrane (IM1, IM2 and IM3).

To further confirm membrane identification NADH oxidase activity was measured in each fraction. The activity of that enzyme should be limited to the inner membrane fractions as this enzyme is located on cytoplasmic side of inner membrane [30]. The highest activity of the enzyme in IM1 and IM2 bands (Fig. 7c) confirmed that the bands belonged to the inner membrane. There was no activity of the enzyme in the additional band (fractions 62–87). It suggests that this band contained cell debris or highly fragmented membranes and probably was not a part of the inner membrane. A low, residual activity of NADH oxidase could also be observed in an OM band. It may be due to the contamination of the outer membrane fractions with inner membrane fractions or incomplete separation of the bands.

1-*N*-fenylnaphtylamine (NPN) is a compound which fluorescence increases in hydrophobic environment after binding to membrane phospholipids [39]. NPN fluorescence is increased only after damaging the outer bacterial membrane and therefore it is used as a probe in outer membrane permeabilization assays [37, 39, 42]. It is due to higher phospholipids content in the inner membrane. Therefore, in this work NPN was used as an inner membrane marker. NPN fluorescence peak was localized only in the IM1 band which further confirmed that the band consisted of inner membrane fractions (Fig. 7d).

Additional identification of the membrane fractions by transmission electron microscopy gave results consistent with the structures observed by others [29, 40, 41]. It is believed that the coiled and C shaped structures are formed due to the chelating effect of EDTA [43]. The binding of divalent cations by EDTA causes disruption of the outer membrane which peels off the cell surface, forming coiled structures.

In order to investigate if the polymer interacts with bacterial membranes leading to their destruction and cell death, the bacterial cells were incubated with fluorescein-labelled polymer (33 µg/ml) and then the membrane fractions were isolated and analysed for polymer fluorescence (Fig. 8). A fluorescein-labelled polymer was located only within the inner membrane fractions. Moreover, the addition of the polymer at this concentration, despite the lethal effect on bacterial cells (MIC = 30 µg/ml), did not cause any noticeable effect on the fraction patterns. It may be explained in two ways. The polymer could first bind to the outer membrane and then pass to the inner membrane without disrupting the outer membrane structure. The polymer could also detach from the outer membrane during membrane preparation procedure and then insert into the inner membrane. It would suggest that the polymer shows greater affinity towards the inner membrane. Alternatively, the concentration of the polymer could be also too small to cause damage to the membranes that could be visualized in membrane separations.

Additional experiments were therefore conducted where the polymer at larger concentrations was added to the earlier isolated “crude” membrane fractions. In this case, the separation patterns were remarkably different in comparison to control without the polymer (Fig. 9). The damaging effect of the polymer was greater on the outer membrane which was visualized as a diminishment of the outer membrane band. A smaller effect the polymer exerted on the inner membrane. The damaging effect on the isolated membranes was concentration dependent.

The observation that the polymer remained bound to the inner membrane in each studied case proves that the affinity of the polymer is much higher to the inner membrane than the outer membrane. It is possibly due to a different lipid composition of the membranes. The outer membrane is asymmetrical. The main component of external leaflet of the outer membrane is LPS. The inner leaflet on the other hand comprises similar lipids as those found in the inner bacterial membrane [44]. The results suggest that the polymer incubated with bacterial cells first attaches to the outer membrane, causing its disruption, which allows it to insert into the inner membrane.

The experiments we have conducted on the water-insoluble analogue immobilized on glass surfaces (data not shown) proved that the water-insoluble polymer was also active against Gram positive and Gram negative bacteria. The polymer showed higher activity against *S. aureus* than *E. coli*, similarly to its water-soluble derivatives. During 40 min. of incubation with the polymer the number of viable *E. coli* bacteria decreased by 5 log CFU/ml. Additionally, SEM analysis confirmed that *E. coli* cells are permanently bound to the polymer surface. The contact with the polymer resulted in formation of blebs in membranes, distortion of cell contours and cell lysis. Moreover, we proved that the polymer inhibits formation of biofilm and causes eradication of already formed *E. coli* biofilm. The practical aspect of these experiments is a potential application of those polymers as antimicrobial coatings and prevention of biofilm formation on surfaces.

Generally, antibacterial polymers tethered on surfaces may act in two ways. They may kill bacteria on contact or at the interface between bacteria and polymer [18]. However, the water-soluble polymers probably work differently than those tethered on surfaces. The damage of the cell in the case of bacteria immobilized on the polymer surface is localized and limited to contact area between the polymer and the cell. Usually, it happens when the density of the active groups on the surface of the polymer is sufficient and adhesion forces are strong. When the polymer is soluble the entire surface of the cell is exposed to the killing factor. It is well visible on transmission electron microscopy micrographs where the sites of the membrane damage are randomly distributed on the whole cell surface (Fig. 6). Hydrophobic tail of the polymers penetrates the membranes on the entire surface of the cell, leading to the full disintegration of the membranes and cell death. In this case the polymer binds to bacterial cells permanently.

The studied polysiloxane-methacrylate copolymer has a methacrylate chain and similar groups to water-insoluble poly[2-(*tert*-butylamino)ethyl methacrylate] polymer (PEB-b-PTBAEMA) studied by Lenoir et al. [25], which may suggest that the polymers have a similar target site. The contact of PEB-b-PTBAEMA with *S. aureus* cells was reversible and the cells were released from the polymer surface. However, polysiloxane-methacrylate copolymer interactions with bacterial cells are stronger, it does not stay bound to the outer membrane and instead inserts into the inner membrane, probably due to stronger hydrophobic interactions with the inner membrane lipids. The mechanism of insertion into the inner membranes may resemble that of antimicrobial random polymers mimicking host-defence peptides such as maganin. The overall antimicrobial activity of such polymers depends more on distribution of lipophilic and cationic groups than the chains identity [45]. That amphiphilic conformation may be induced within the polymer in contact with bacterial surface. It may be presumed that similar amphiphilic conformation may be also induced in our copolymers. The presence of elastic polysiloxane chain, which can easily rotate round Si–O bond, makes it easier for ionic groups to reorientate within the polymer. The association with the membranes may then proceed via a mechanism similar to the “carpet” mechanism [46, 47]. Alternatively, the membrane disruption may be a result of aggregation of charged lipids and subsequently generation of defects between lipid domains [32].

## Conclusions

Potential wide applications in medicine and industry of antibacterial polysiloxane-methacrylate copolymers with pending *t*-butylammonium groups are related to their high antimicrobial activity. The binding of the polymer to bacterial superficial structures causes changes in bacterial cells morphology and structure, and leads to changes in bacterial membranes permeability. The polymers have different affinity to membranes of Gram negative bacteria, exerting more destructive effect on the outer membrane.
